# Quaternized Chitosan as an Antimicrobial Agent: Antimicrobial Activity, Mechanism of Action and Biomedical Applications in Orthopedics

**DOI:** 10.3390/ijms14011854

**Published:** 2013-01-16

**Authors:** Honglue Tan, Rui Ma, Chucheng Lin, Ziwei Liu, Tingting Tang

**Affiliations:** 1 Shanghai Key Laboratory of Orthopedic Implants, Department of Orthopedic Surgery, Shanghai Ninth People’s Hospital, Shanghai Jiao Tong University School of Medicine, Shanghai 200011, China; E-Mails: hnlc.love@163.com (H.T.); shandongmarui@126.com (R.M.); 2 The Key Laboratory of Inorganic Coating Materials, Chinese Academy of Sciences, Shanghai 200011, China; E-Mails: chucheng@mail.sic.ac.cn (C.L.); ziweiliu@mail.sic.ac.cn (Z.L.)

**Keywords:** quaternized CS, antimicrobial activity, mechanism, application, orthopedics

## Abstract

Chitosan (CS) is a linear polysaccharide with good biodegradability, biocompatibility and antimicrobial activity, which makes it potentially useful for biomedical applications, including an antimicrobial agent either alone or blended with other polymers. However, the poor solubility of CS in most solvents at neutral or high pH substantially limits its use. Quaternary ammonium CS, which was prepared by introducing a quaternary ammonium group on a dissociative hydroxyl group or amino group of the CS, exhibited improved water solubility and stronger antibacterial activity relative to CS over an entire range of pH values; thus, this quaternary modification increases the potential biomedical applications of CS in the field of anti-infection. This review discusses the current findings on the antimicrobial properties of quaternized CS synthesized using different methods and the mechanisms of its antimicrobial actions. The potential antimicrobial applications in the orthopedic field and perspectives regarding future studies in this field are also considered.

## 1. Introduction

Chitosan (CS), as a polycationic polymer, is obtained from crustacean shells by partial and full alkaline deacetylation [1]. As a result of its biodegradability, nontoxicity and antimicrobial activity, CS has been widely used for biomedical applications, such as tissue engineering scaffolds, drug delivery, wound dressings and antibacterial coatings [1,2]. However, CS is insoluble in most solvents at neutral or high pH, except in organic acids, which substantially limits its usefulness [1]. To address this limitation, CS derivatives by chemical modifications have recently been studied. One purpose of the chemical modifications of CS, which have included saccharization, alkylation, acylation, quaternization and metallization, has been to improve its water solubility and increase its antimicrobial activity. As one example, quaternary ammonium CS can be prepared by introducing a quaternary ammonium group on a dissociative hydroxyl group or amino group. In some studies, quaternized CS derivatives exhibited stronger antibacterial activity, a broader spectrum and higher killing rates compared to unmodified CS [3–7].

Based on the current state of research and progress in corresponding areas, this review attempts to summarize the antimicrobial properties of quaternized CS synthesized with different methods and modes of action as antimicrobial compounds. Subsequently, the present and potential future applications of this material in the orthopedic field are also discussed in detail.

## 2. Antibacterial Activity of Quaternised CS

Quaternised CS, which introduces permanent positively charged quaternary ammonium groups and enhances water solubility, has attracted considerable attention recently as an antibacterial agent over a broad pH range [8–10]. In recent series studies, the synthesis and characterization of different quaternized CS derivatives were described [5–7,11–17].

N-substituted CS was quaternized using *N*-(3-chloro-2-hydroxy-propyl) trimethylammonium chloride (GTMAC) to increase its water solubility. The minimum inhibitor concentration (MIC) experiment was performed on *E. coli* and *S. aureus* to explore the impact of the extent of N-substitution (ES) on the antibacterial activities. The results showed that when the ES is higher than 20%, MIC values are also higher. The antibacterial activities ranged from 8 to 64 μg/mL for *S. aureus* and from 16 to 64 μg/mL for *E. coli* [13]. Another study from the same research group presented N-methylation of N-arylated CS derivatives containing *N*,*N*-dimethylaminophenyl and pyridyl substituents, which produced quaternary ammonium salts in the presence of sodium iodide and iodomethane. The methylated products were water soluble over all pH ranges and displayed antibacterial activity against *S. aureus* and *E. coli*. Their MIC values were in the range of 32–128 μg/mL against both bacteria [14]. A di-quaternary group can contribute to the antibacterial activity of CS; an obvious inhibition against the G^+^ strains of *S. aureus* and *Staphylococcus pneumoniae* was found by a CS derivative with an *N*-[1-carboxymethyl-2-(1,4,4-trimethylpiperazine-1,4-diium)] substituent, which was generally more active at pH 7.2 than at pH 5.5 [15]. Quaternary ammonium and disaccharide CS were successfully synthesized by using *N*-(3-chloro-2-hydroxypropyl) trimethylammonium chloride (GTMAC) as a quaternizing agent to modify residual free primary amino groups and some hydroxyl groups of CS derivatives. All quaternary ammonium CS derivatives were water-soluble over an entire range of pH values and displayed antibacterial activity against *S. aureus* and *E. coli*, as observed by using the MIC method. The results implied that *N*-benzyl chitosans GTMAC derivatives would be useful as potential new antibacterial agents [5]. The permanent positive charges in the form of quaternary ammonium groups were introduced to the surface of pre-fabricated CS particles under heterogeneous conditions via either a direct methylation or a reductive N-alkylation using two different aldehydes—propionaldehyde and benzaldehyde—followed by methylation with methyl iodide. It was found that all quaternized CS particles exhibited a higher antibacterial activity against *S. aureus* than the CS particles did in a neutral pH media. The results obtained from this research suggest that the surface-quaternized CS particles may potentially be used as an effective antibacterial material for biomedical applications [16].

Numerous studies support the essential importance of a polycationic structure in antimicrobial activity. The positive charge is associated with the degree of substitution (DS) of CS derivatives, which affect positive charge density [17]. With regard to CS derivatives, antimicrobial activity mostly depends on the DS of the grafting groups. With a different degree of substitution of the quaternary ammonium, quaternized CS exhibits different antibacterial activities. *N*,*N*,*N*-trimethyl *O*-(2-hydroxy-3-trimethylammonium propyl) chitosans (TMHTMAPC) with different degrees of *O*-substitution were synthesized by reacting *O*-methyl-free *N*,*N*,*N*-trimethyl CS (TMC) with 3-chloro-2-hydroxy-propyl trimethylammonium chloride (CHPTMAC). This quaternized CS exhibited enhanced antibacterial activity compared with TMC alone, and the activity increased with an increase in the degree of substitution [7]. Hydroxypropyltrimethylammonium chloride CS (HACC) was synthesized with differing degrees of substitution (6%, 18% and 44%) of quaternary ammonium by reacting CS with glycidyl trimethylammonium chloride. The antibacterial activities of these polymers were tested *in vitro* against *Staphylococcus aureus*, Methicillin-resistant *Staphylococcus aureus* and *Staphylococcus epidermidis*. The results showed that the antibacterial activities of the HACC with 18% or 44% substitution were significantly higher than the HACC with 6% substitution or CS alone against all three bacteria [6].

However, there exist discrepancies among different reports on the antibacterial activity of CS and its quaternary derivatives. *N*,*N*,*N*-diethylmethyl CS exhibits enhanced antibacterial activity against *E. coli* in comparison with CS and a decrease in pH results in stronger activity [18]. HTCC, which was prepared by the reaction of CS with GTMAC, displays an increased antibacterial efficiency against both *E. coli* and *S. aureus* relative to CS [19,20]. In contrast, Chi *et al.* [21] found that HTCC had no notable activity against *E. coli*. Qin *et al.* [22] reported that the antibacterial activity of HTCC was stronger under alkaline conditions than under weak acidic conditions. In a previous report, modification of the free amino group of the CS backbone decreased its antibacterial activity [23]. These results showed that quaternization does not always enhance the antibacterial activity of CS and that the effect of pH on the antibacterial activity of quaternary ammonium CS is uncertain. The discrepancies among different reports on the antibacterial activity of CS and its quaternary derivatives are most likely caused by various intrinsic and extrinsic factors that are related to the CS itself (e.g., type, MW, DD, viscosity solvent and concentration) and the environmental conditions (e.g., test strain, its physiological state and the bacterial culture medium, pH, temperature, ionic strength, metal ions and organic matter), respectively [24].

In addition, the biocompatibility of the quaternized CS should be considered. Mouse fibroblasts and bone-marrow-derived stromal cells (hMSCs) were used to investigate the biocompatibility of the HACC. The results showed that the antibacterial activities of the HACC with a higher degree of substitution of the quaternary ammonium were significantly higher than the lower degree of substitution against bacteria. However, they also exhibited interference in the proliferation and osteogenic differentiation of hMSCs and a cytotoxic effect on the activities of the mouse fibroblasts. Conversely, HACC with a lower substitution was highly biocompatible with osteogenic cells [6].

Based on the above analysis, the antibacterial activity of quaternized CS has been shown to be stronger than that of CS, and the antibacterial activity increases accordingly with the increasing DS of the quaternary ammonium. However, the biocompatibility of the quaternized CS was affected with the different DS, with higher DS exhibiting a cytotoxic effect on the cell activity. In addition, there existed discrepancies among the antibacterial activities of CS and its quaternary derivatives. Therefore, further investigation should be performed.

## 3. Antifungal Activity of Quaternised CS

The modification of CS to improve its activity is a promising approach to achieving effective bio-fungicides [15,25]. With the development of the science of CS, it has been found that CS has antifungal activities [1,26]. Based on the available evidence, bacteria appear to be generally less sensitive to the antimicrobial action of CS than fungi [17]. EI Ghaouth reported that CS could inhibit the growth of *Alternaria alternata*, *Botrytis cinerea*, *Colletotrichum gloeosporioides* and *Rhizopus stolonifer* [27]. The growth of fungi, such as *F. oxysporum*, *R. stolonifer*, *Penicillium digitatum* and *C. gloeosporioides* can be completely inhibited by CS at a concentration of 3% [28–30]. Four CS types with different MWs were tested against fungal pathogens. The most bioactive type of CS that inhibited the growth of *Candida albicans* had the lowest molecular weight (32 kDa) and the highest degree of deacetylation (94%). The MIC of this CS type towards *C. albicans* strains were 2.0, 1.75 and 1.25 mg/mL against *C. albicans-A*, *C. albicans-H* and *C. albicans-C*, respectively [31]. However, relatively little work has been reported on the antifungal activities of quaternized CS derivatives.

Quaternised chitosans—including *N*-(2-hydroxyl-phenyl)-*N*, *N*-dimethyl CS (NHPDCS), *N*-(5-chloro-2-hydroxyl-phenyl)-*N*, *N*-dimethyl CS (NCHPDCS), *N*-(2-hydroxyl-5-nitro-phenyl)-*N*, *N*-dimethyl CS (NHNPDCS) and *N*-(5-bromic-2-hydroxyl-phenyl)-*N*,*N*-dimethyl CS (NBHPDCS)—were synthesized and their antifungal activities against *Botrytis cinerea Pers*. (*B. cinerea Pers*.) and *Colletotrichum lagenarium (Pass) Ell. et Halst* (*C. lagenarium (Pass) Ell. et Halst*) were investigated. The results indicated that all the quaternized CS derivatives have better antifungal activities compared with the activity of unmodified CS [32]. In another study, quaternized CS derivatives with different molecular weights were synthesized, their antifungal activities against *Botrytis cinerea Pers*. (*B. cinerea Pers*.) and *Colletotrichum lagenarium (Pass) Ell. et Halst* (*C. lagenarium (Pass) Ell. et Halst*) were conducted. The results indicated that quaternized chitosan derivatives have stronger antifungal activities than chitosan. Furthermore, quaternized chitosan derivatives with high molecular weight were shown to have even stronger antifungal activities than those with low molecular weight [33].

Two series of new quaternized CS derivatives were synthesized by the reaction of deacetylated chitosan (CH) with propyl (CH-Propyl) and pentyl (CH-Pentyl) trimethylammonium bromides to obtain derivatives with increasing degrees of substitution (DS). The antifungal activities of these derivatives on the mycelial growth of *Aspergillus flavus* were investigated *in vitro*. The results showed that the antifungal activities increased with DS and that the more substituted derivatives of both series, CH-Propyl and CH-Pentyl, exhibited antifungal activities three and six times higher, respectively, than those obtained with commercial and deacetylated CS. The results showed that the quaternary derivatives inhibited the fungus growth at one-fourth the polymer concentration of deacetylated chitosan (CH). The antifungal activity of CS can be improved by increasing the degree of substitution (DS) of alkyltrimethylammonium groups on the polymer chain. The inhibition indexes for both synthesized series (propyl and pentyltrimethylammonium) increased with the DS, and the most substituted derivatives (CH-Propyl-40 and CH-Pentyl-65) exhibited inhibition values three and six times higher, respectively, than those obtained with CS [34].

Five water-soluble chitosan derivatives were recently carried out by quaternizing either iodomethane or GTMAC as a quaternizing agent under basic condition [35]. The degree of quaternization (DQ) ranged between 28% ± 2% and 90% ± 2%. The antifungal activity was evaluated by using the disc diffusion method, MIC and minimum fungicidal concentration (MFC) methods against *Trichophyton rubrum* (*T. rubrum*), *Trichophyton mentagrophyte* (*T. mentagrophyte*) and *Microsporum gypseum* (*M. gypseum*) at pH 7.2. All quaternized chitosans and its derivatives were shown to be more effective against *T. rubrum* than *M. gypseum* and *T. mentagrophyte*. The MIC and MFC values were found to range between 125–1000 μg/mL and 500–4000 μg/mL, respectively, against all fungi. The results indicated that the quaternized *N*-(4-*N*,*N*-dimethylaminocinnamyl)chitosan chloride showed highest antifungal activity against *T. rubrum* and *M. gypseum* compared to other quaternized chitosan derivatives. The antifungal activity tended to increase with an increase in molecular weight, degree of quaternization and hydrophobic moiety against *T. rubrum*. However, the antifungal activity was dependent on type of fungus, as well as the chemical structure of the quaternized chitosan derivatives.

In summary, quaternized CS derivatives have better antifungal activities than that of unmodified CS. The antifungal activity of CS can be improved by increasing the degree of substitution (DS) of the quaternary ammonium of the CS. However, the investigation of the antifungal activity of the quaternized CS was not performed in clinically derived fungi.

## 4. The Mechanism of Antimicrobial Action

### 4.1. Mechanism of Antibacterial Action

The exact mechanisms of the antibacterial activities of CS or quaternized CS are still unknown. The polycationic structure of quaternized CS is a prerequisite for antibacterial activity. Electrostatic interaction between the polycationic structure and the predominantly anionic components of the microorganisms play a fundamental role in antibacterial activity. The number of ammonium groups linking to the CS backbone is important in electrostatic interaction for the antibacterial activity of quaternized CS. It has been reported that quaternized CS with a higher degree of substitution of the quaternary ammonium exhibited a strong interaction with negative charges on the bacterial cell surface and showed better antibacterial activity than CS [5,7,17,36].

The additional effect derived from the hydrophobic–hydrophobic interactions between the aryl substituent and the hydrophobic interior of the bacterial cell wall is proposed to explain the mechanisms of the antibacterial activities, because alkyl substituents with an increased chain length on the quaternary ammonium CS salt also displayed higher antibacterial activities [13,37]. The hydrophobicity and cationic charge density of the introduced substituent play important roles in determining the antibacterial activity of quaternized CS derivatives. In addition, under neutral or higher pH, quaternized CS modified with lipophilic groups showed higher activity than native CS, and the hydrophobic and chelating effects may be responsible for antibacterial activity in addition to the electrostatic effect [17].

The antibacterial action of the CS derivatives is also based on the physical states and molecular weight. In general, high-molecular-weight CS derivatives and solid particles cannot pass through cell membranes and only interact with the cell surface to alter cell permeability [38] or form a film around the cell that protects cells against nutrient transport through the microbial cell membrane [39]. However, low-molecular-weight, water-soluble CS derivatives or nanoparticles could penetrate the cell walls of bacteria, incorporate with DNA and inhibit the synthesis of mRNA and DNA transcription [40].

The microorganism may also affect the antimicrobial activity of the quaternized CS. The quaternized *N*-aryl CS derivatives were not as effective against *E. coli* bacteria as against *S. aureus* bacteria [41], because the outer membrane (OM) of Gram-negative bacteria functions as an efficient barrier against macromolecules, such as CS derivatives. The OM, which contains lipopolysaccharide (LPS), provides the bacterium with a hydrophilic surface. The lipid components and the inner core of the LPS molecules contain anionic groups that contribute to the stability of the LPS layer through electrostatic interactions with divalent cations [42]. Therefore, overcoming the OM is a prerequisite for any material to exert bactericidal activity towards Gram-negative bacteria [43,44]. There exists, however, a converse viewpoint. Polyanions on the cell surface take part in the electrostatic interactions with CS derivatives. The negative charge on the cell surface of the tested Gram-negative bacteria (*Pseudomonas aeruginosa, Salmonella typhimurium and Escherichia coli*) was higher than on the tested Gram-positive bacteria (*Staphylococcus aureus* and *Streptococcus faecalis*), leading to more CS derivatives adsorbed and higher inhibitory effects against the Gram-negative bacteria [45].

Despite the distinction between Gram-negative and Gram-positive bacterial cell walls, antibacterial modes both begin with interactions at the cell surface and compromise the cell wall or OM first. For Gram-positive bacteria, lipoteichoic acids may provide a molecular linkage for CS derivatives at the cell surface, allowing it to disturb membrane functions [46]. LPS and proteins in the Gram-negative bacteria OM are held together by electrostatic interactions with divalent cations that are required to stabilize the OM. Polycations may compete with divalent metals, such as Mg^2+^ and Ca^2+^ ions present in the cell wall, which will disrupt the integrity of the cell wall or influence the activity of degradative enzymes [17]. Contact between CS derivatives and the cell membrane, which is essentially a negatively charged phospholipid bilayer, may slightly change the membrane permeability. Further interactions may denature membrane proteins and initiate penetration into the phospholipid bilayer. The increased membrane permeability leads to destabilization of the cell membrane, leakage of intracellular substances and, ultimately, the death of cells [17,47,48].

### 4.2. Mechanism of Antifungal Action

Similar to the antibacterial action, the antifungal activity of CS derivatives is believed to occur from the interaction between the cationic chain and the negatively charged residues of macromolecules exposed on the fungal cell surface, leading to a leakage of intracellular electrolytes and other constituents [49–53]. It is believed that CS may affect the morphogenesis of the cell wall, interfering directly with the activity of enzymes responsible for growth of the fungi [27]. Recently, Li *et al.*, based on confocal laser scanning microscopy of fluorescein-labeled chitosans, showed that low-molecular-weight chitosans could enter into the hypha of *Fulvia fulva*, suggesting that the growth of *F. fulva* may be inhibited by chitosans from inside the cell [54]. The target site of the cation is the negatively charged cell surface membrane, which prevents nutrients from entering the cell, and the antifungal activity of quaternized CS is also likely the result of this type of activity [32].

Another possibility for the antifungal activity of CS is based on its chains crossing the cell membrane, inhibiting the cell from growing from the inside [32]. However, for deacetylated chitosan (CH) and the derivatives with low degrees of substitution, the mechanism on *A. flavus* and the interaction with the cell surface may form an impermeable layer around the cell, thus blocking the transport of essential solutes into the cell [39]. The modification of CS by introducing permanently charged quaternary groups may improve the antifungal activity of CS. The addition of quaternized CS to the BDA medium inhibited the mycelial growth of *A. flavus* significantly at all concentrations tested. The results obtained in microbiological assays showed that the capability to inhibit fungus growth *in vitro* was clearly increased for the higher degrees of substitution for the two series tested [39,54].

### 4.3. Mechanism of Anti-Biofilm Formation

Implant-associated infection is primarily caused by bacterial growth in biofilms [55]. Biofilm formation is considered to be an important virulence mechanism, because the biofilm impairs the activity of antibiotics, prevents normal immune responses and complicates the eradication of infections [56,57]. Once an infection has been established and a well-organized biofilm has formed on the implant surface, antibiotic therapies are less efficacious and removal and substitution of the implant are often the only way to eradicate the problem [58,59]. Bacterial adherence to orthopedic implant surfaces occurs in two essential steps [60]. Its adherence to the implanted surface is followed by an accumulation process and the production of extracellular substances, such as polysaccharide intercellular adhesin (PIA) [61,62]. The production of PIA is mediated by the intercellular adhesin (ica) locus, which comprises four core genes (*ica*A, *ica*B, *ica*C and *ica*D) and a regulatory gene (*ica*R) [63–65]. Peng ZX, *et al.* [66,67] assessed *ica*A transcription as an index of biofilm formation on a titanium surface by RT-PCR. The results showed that HACC can inhibit *ica*A expression and the level of inhibition increased with higher HACC concentrations in the biofilm prevention and susceptibility assays. This effect was more significant for HACC concentrations of 18%, 26% and 44%, which blocked the transcription of *ica*A at concentrations of 128 μg/mL and 256 μg/mL in the biofilm prevention assay and at 256 μg/mL in the biofilm susceptibility assay. The authors postulated that this inhibition of *ica*A transcription results in reduced biofilm formation and increased susceptibility to HACC, because the biofilm protects and supports the growth of bacteria on the surface of implants and inhibition of biofilm formation further impairs bacterial viability. This transcriptional response data may provide indirect evidence that quaternized CS treatment interferes with cellular energy metabolism.

## 5. Application in the Orthopedic Surgery Field

Biomaterial-associated infections remain a serious complication in orthopedic surgery. Antimicrobial prophylaxis, including the systemic and local use of antibiotics, has proven to be valuable in the prevention of infection in both experimental and clinical research [68–70]. Using bone cement as the carrier for antibiotics is a way of delivering high levels of antibiotics locally without causing systemic toxicity. PMMA bone cements and beads loaded with gentamicin are becoming the standard practice for preventing infection in joint arthroplasty and for treating infection in osteomyelitis [71–74]. However, the overuse of antibiotics leads to the evolution of antibiotic-resistant bacteria, especially methicillin-resistant *Staphylococcus aureus* (MRSA) and methicillin-resistant *Staphylococcus epidermidis* (MRSE) [70,75]. In addition, to succeed in orthopedic surgery, implant materials must be anti-infective (discouraging bacterial adhesion), as well as habitable by bone-forming cells (favoring the activity of osteogenic cells) [76,77]. According to previous studies, gentamicin at high local concentrations reduced the viability, proliferation and alkaline phosphatase activity of the osteoblasts [78–83] and inhibited the proliferation and differentiation of human bone marrow mesenchymal stem cells *in vitro* and *in vivo* [78,84,85], which compromised the bone-healing process. To overcome these disadvantages derived from antibiotics, a new quaternized CS derivative (hydroxypropyltrimethylammonium chloride CS, HACC) with a 26% degree of substitution was synthesized and loaded at a 20% by weight ratio into PMMA bone cement to investigate whether HACC in PMMA prevents bacterial biofilm formation on the surface of bone cements. Two clinical isolates, *Staphylococcus epidermidis* 389 and methicillin-resistant *S. epidermidis* (MRSE 287) and two standard strains, *S. epidermidis* (ATCC 35984) and methicillin-resistant *Staphylococcus aureus* (ATCC 43300), were selected. The results showed that HACC-loaded PMMA inhibited biofilm formation on its surface compared to other control groups, providing a promising new strategy for combating implant infections and osteomyelitis [66]. The same research team simultaneously found better stem cell proliferation, osteogenic differentiation and osteogenesis-associated gene expression on the surface of the HACC-loaded PMMA compared to the gentamicin-loaded PMMA. Therefore, this new anti-infective bone cement also exhibited improved physical properties and osteogenic activity, which may lead to better osseointegration of the bone cement in cemented arthroplasty [86]. Shi ZL *et al.* produced similar results. In their research, the use of CS nanoparticles (CS NP) and quaternary ammonium CS derivative nanoparticles (QCS NP) as bactericidal agents in poly(methyl methacrylate) (PMMA) bone cement with and without gentamicin were investigated. The antibacterial activity was tested against *S. aureus* and *S. epidermidis*. This *in vitro* study demonstrated that the incorporation of nanoparticles of CS and quaternary ammonium CS derivative in bone cements can provide effective antibacterial action against *S. aureus* and *S. epidermidis*. These nanoparticles also enhance the antibacterial efficacy of gentamicin-loaded bone cements, and this property is retained even after an extended period of immersion of the modified bone cement in an aqueous medium [87].

Titanium-based biomaterials are currently the best and most widely used materials in the manufacture of orthopedic and dental implants, because of their high strength, low weight, excellent corrosion resistance and good biocompatibility [88–90]. The biocompatibility of titanium implants can be attributed to a surface protein layer formed under physiological conditions that actually makes the surface suitable for bacterial colonization and biofilm formation [91–93]. The long-term success of orthopedic implants may be compromised by defective osseointegration and bacterial infection. To overcome these two major problems of Ti implants, an effective approach to minimizing implant failure would be to modify the surface of the implant to make it habitable for bone-forming cells and anti-infective at the same time [94–98]. In one study, the efficacy of hydroxypropyltrimethylammonium chloride CS (HACC) with different degrees of substitution (DS; referred to as HACC 6%, 18% and 44%) in preventing biofilm formation on a titanium surface was evaluated [67]. The results showed that HACC, especially HACC with DS of 18% and 44%, significantly inhibited biofilm formation compared to the CS control, even at concentrations far below their MICs. Therefore, HACC may serve as a new antibacterial agent to inhibit biofilm formation and prevent orthopedic implant-related infections. In addition, HACC with DS of 18% exhibited good biocompatibility with osteogenic cells, which is beneficial to the osseointegration of the implants [6].

In orthopedic surgery, open fractures with bacterial infections are often seen and the treatment of these injuries is challenging for surgeons. Biomaterials with properties that promote wound healing and simultaneously eliminate infections have attracted the interest of scientists. Chitosans, which have hydrogel-forming properties, have been considered to be advantageous in their application as a wound dressing material, and CS-based materials have received attention in this regard [99–103]. A majority of micro- and nano-fiber materials derived from antimicrobial products are suitable for preparing wound dressings. Electrospinning is a favorable technique for producing continuous polymer fibers with diameters down to the nanoscale range [104]. Because of unique properties, such as their high surface-to-volume ratio, high porosity and diameters at the nanoscale, electrospun mats made from ultrafine polymer fibers have been drawing great interest. Quaternized CS has shown high antibacterial activity against Gram-positive and Gram-negative bacteria. In one study, quaternized CS-containing nanofibers were successfully prepared by the electrospinning of mixed aqueous solutions of QCh and PVA, and the electrospun QCh/PVA (quaternized chitosan/poly vinyl pyrrolidone) nanofibrous mats were efficient in inhibiting the growth of Gram-positive bacteria and Gram-negative bacteria [4]. In addition, in this authors’ previous work [3], the antibacterial activity of cross-linked electrospun QCh/PVA (polyvinyl alcohol) mats made of quaternized CS derivatives was observed to be bactericidal rather than bacteriostatic. PVA are nontoxic, biocompatible and highly hydrophilic, while possessing good complexation properties and good film-forming abilities. Therefore, the antibacterial activity of electrospun QCh/PVA mats is an important property for wound-healing applications, because it has the potential to contribute to the prevention of secondary infections in wounds by *S. aureus*, resulting in limited scar formation [3,4,105].

## 6. Perspectives and Areas for Future Research

Although quaternized CS derivatives have been regarded as effective antimicrobial agents, their modes of action need to be further studied in depth. Investigations have recently focused on the morphological changes of the microorganism with respect to its antimicrobial property and mechanism. The methods used to evaluate the antimicrobial phenomena have been limited to the biological conception only. Therefore, future work should aim at demonstrating the molecular details of the underlying mechanisms and their relevance to the antimicrobial activity of quaternized CS. Additionally, whether this compound induces bacterial resistance and its mechanisms should also be considered.

According to previous reports, with the increasing DS of the ammonium of the quaternized CS, the antimicrobial activities were enhanced, but the cytotoxicity also increased. Thus, the toxicity and biocompatibility of this CS derivative should be the main focus of further studies. Finding the DS with a balance between good antimicrobial activity and low mammalian toxicity is important.

Studies about this compound have largely been performed in *in vitro* experiments. It is important to clarify its potential use as an antimicrobial agent *in vivo*. In that sense, researchers should emphasize *in vivo* studies to establish the efficacy of the antimicrobial activity of quaternized CS. Furthermore, quaternized CS would be valuable as an effective antibacterial coating or antimicrobial dressing in orthopedic surgery. Therefore, a significant increase in the number of scientific studies to obtain evidence to support this use can be expected.
